# Neural Control of Stopping and Stabilizing the Arm

**DOI:** 10.3389/fnint.2022.835852

**Published:** 2022-02-21

**Authors:** Shanie A. L. Jayasinghe, Robert A. Scheidt, Robert L. Sainburg

**Affiliations:** ^1^Department of Neurology, Pennsylvania State University College of Medicine, Hershey, PA, United States; ^2^Department of Biomedical Engineering, Marquette University and Medical College of Wisconsin, Milwaukee, WI, United States; ^3^Department of Kinesiology, Pennsylvania State University, State College, PA, United States; ^4^Huck Institutes of the Life Sciences, Pennsylvania State University, State College, PA, United States

**Keywords:** muscle, impedance control, upper limb, motor control, movement

## Abstract

Stopping is a crucial yet under-studied action for planning and producing meaningful and efficient movements. In this review, we discuss classical human psychophysics studies as well as those using engineered systems that aim to develop models of motor control of the upper limb. We present evidence for a hybrid model of motor control, which has an evolutionary advantage due to division of labor between cerebral hemispheres. Stopping is a fundamental aspect of movement that deserves more attention in research than it currently receives. Such research may provide a basis for understanding arm stabilization deficits that can occur following central nervous system (CNS) damage.

## Introduction

When examining the neural mechanisms that underlie control of upper limb movements in humans, previous research has predominantly focused on how the nervous system specifies and actuates movement trajectories. However, an underappreciated yet critical aspect of motor control is the ability to stop movement at an intended and stable position, such as when a tennis player runs up to the net, stops, and hits a drop shot without falling through the net. Complex, graceful behaviors require that maneuverability of action be complemented with the ability to stabilize the body rapidly and precisely, and biological impedance control provides an elegant solution to the stopping problem.

Consider what would be needed to bring the hand to rest at a target without a specialized mechanism for controlling impedance. Precise joint torques would need to be planned and applied to decelerate a given motion, and inaccuracies due to “noise” and inaccurate predictions in motor commands and/or delays in sensory feedback would inevitably lead to instability. The arrangement of agonist and antagonist muscles across our joints allows two types of braking mechanisms—deceleration through the activation of task antagonists (imagine a car screeching to a halt at a traffic light), and position- and velocity-dependent impedance control through coactivation of agonist and antagonist muscles (similar to air brakes on an airplane that are used to reduce drag but not lift).

Mechanical impedance relates forces to resulting motions (velocities). As such, impedance can be characterized by the inertial, viscous, and elastic resistance to motion. Limb stiffness and viscosity can be modulated through muscle coactivation (i.e., simultaneous activation of opposing muscles at a joint) (Hogan, 1984; Lacquaniti et al., 1993) and through the modulation of proprioceptive reflex gains and thresholds (Takahashi et al., 2001; Pruszynski and Scott, 2012; Sainburg, 2014). Limb endpoint inertia is configuration-dependent (Hogan, 1985), and stiffness modulation *via* synergistic activation of muscles is used for postural coordination (Mussa-Ivaldi et al., 1985). We propose that the action of stopping requires defining a specific limb configuration, and also defining parameters such as viscosity and stiffness.

## How We Move Depends on How We Stop—And *Vice Versa*

There are two distinct ways in which movements can be “stopped.” In one, inhibitory circuits in the brain suppress activity in cortical structures involved in movement planning and execution prior to the initiation of the movement (Nielson et al., 2002; Lemon and Kraskov, 2019). Inhibitory control is an aspect of executive functioning that is a focus of much research on decision making in health, normal aging, and disease (Rubia et al., 2001; Aron et al., 2007; Langenecker et al., 2007; Votruba et al., 2008; Mannarelli et al., 2020; Elverman et al., 2021). In this review, we limit our consideration to the second type of stopping—physical interaction between the motor periphery and the environment, that brings the limb to rest at a new stable posture after a movement has been initiated (c.f. Noorani and Carpenter, 2017).

Early studies of muscle activity during goal-directed reaching have demonstrated the role of antagonist muscle activity in stopping a ballistic movement. Studies of the classic triphasic electromyography (EMG) pattern commonly observed during fast goal-directed movements have identified notable stereotyped behaviors (Hallett et al., 1975; Ghez and Martin, 1982; Meinck et al., 1984; Hannaford and Stark, 1985). Specifically, at the beginning of a movement, any tonic activity in the functional antagonist ceases and a burst of activity in the functional agonist accelerates the limb toward its target. Around the time of peak movement velocity, the initial agonist burst ceases and antagonist activity rises to decelerate the limb. Shortly after the onset of the antagonist, agonist activity again arises as the limb is brought to rest at its intended target (Hallett et al., 1975).

In another seminal study examining the processes that underly transitions from movement to posture, Lestienne et al. (1981) examined the EMG patterns associated with a large range of single joint movements of various amplitudes, speeds, and directions made throughout the range of motion of the elbow and wrist joints. Their findings revealed reciprocal agonist-antagonist EMG patterns that characterized the early phase of motion, followed by coactivation patterns that characterized the later phase of movement. Coactivation patterns extended throughout the deceleration phase of movement into the postural stabilization phase and were characterized by a unique ratio of agonist to antagonist activity that varied with each final posture, a finding that could be explained partially by the differing muscle mechanical states associated with each final limb configuration. The fact that coactivation ratios varied directly with posture, but not with trajectory features such as movement direction, amplitude, or velocity led the authors to conclude: “The motor processes controlling final position and trajectory seem to be independent.” These findings and conclusion support our hypothesis of independent mechanisms for control of trajectory and posture, which will be discussed later in this paper.

Studies with peripherally deafferented individuals have also shown the presence of the triphasic EMG pattern; however, the magnitude of the antagonist burst is not as strongly correlated to the magnitude of the first agonist burst as it is in neurologically intact individuals (Forget and Lamarre, 1987). Importantly, (Brown and Cooke, 1990) showed that humans can modify the triphasic EMG pattern when producing movements with different temporal profiles, such that the duration of initial agonist burst offset and antagonist burst onset can be modified. These studies show that online peripheral feedback is crucial for modulating the limb’s mechanical interaction with the environment through the coordinated activity of functionally antagonistic muscles, and that neither peripheral mechanics nor central commands alone suffice.

In fact, numerous studies have demonstrated that the human central nervous system (CNS) is adept at achieving movement goals despite environmental changes impacting performance. This ability is facilitated by a phenomenon known as sensorimotor adaptation, which is a form of learning whereby the CNS adjusts motor behavior to restore performance in the presence of altered environmental conditions. Adaptation studies further highlight the importance of central influences on how the limb interacts physically with the environment. For example, when a ball is dropped from a specific height, we are able to infer an accurate time of interception and to modulate limb impedance accordingly (Lacquaniti et al., 1993); however, in conditions of reduced gravity, such as during space flight, the timing of interception is inaccurate because visual cues about the target are combined with an *a priori* model of the earth’s gravitational acceleration (Lacquaniti et al., 2015). Adaptation also explains how we experience aftereffects from changing mechanical conditions. For example, when spending time on a boat, the CNS adapts to the rocking of the boat by implementing predictive and reactive mechanisms. Upon returning to stable ground, individuals experience “sea legs,” which is the body countering the swaying that occurred on the boat to maintain balance, and reflects the CNS predicting waves that no longer exist. After a short time, however, this aftereffect goes away, due to readaptation to stable conditions. Such aftereffects have been shown in motor learning studies, and they reflect predictions of previously applied and adapted forces (Lackner and Dizio, 1994; Shadmehr and Mussa-Ivaldi, 1994; Sainburg and Kalakanis, 2000). A recent study showed that humans adapt their movement at different rates depending on the type of load placed on the upper limb—inertial, viscous, elastic—and that such adaptation may be explained by the existence of internal models of limb mechanics that are updated at different speeds (Oh et al., 2021). These studies have been interpreted to reflect a process that models the applied environment dynamics in order to control movements through predictive mechanisms involving neural structures widely distributed throughout the CNS (c.f. Scheidt et al., 2012). The study of central influence on peripheral interactions with the environment continues to be an important topic of research.

## What We Have Learned From Engineered Systems

Various computational models have been used to describe the neural control of movement and stopping. The CNS receives feedback from multiple sources, which suggests that it must decide how and when to integrate different types of feedback for movement planning. Limb movement can be stabilized through the use of neural feedback loops involving the CNS and muscles (Suminski et al., 2007), as well as through predictive control of mechanical impedance in the muscle (Burdet et al., 2001) and multi-articular limb (Hogan, 1985; Mah, 2001). A disadvantage of pure feedback control is that sensory information processing delays can be slow, leading to long loop delays. Therefore, in order to survive, we must be able to plan feedforward commands that are based on internal models (i.e., expectations) of the dynamic interactions between the limb and its environment and their sensory consequences. However, feedforward control also has limitations, including the inability to compensate in real-time for performance errors induced by environmental uncertainties, prediction errors, and potential noise in the motor execution system. Some combination of feedforward and feedback control schemes could have functional utility. How might this work?

One possibility is captured by the idea of model-free control, which has been used to describe biological movement. The equilibrium point hypothesis (Asatryan and Feldman, 1965) proposes that the CNS need not account for or control biomechanical nor environmental dynamics, but instead purports that centrally specified reference configurations (equilibrium positions or “set points”) are a product of coactivation and reciprocal commands that result in stiffness about specified or “referent” configurations. Shifts in the referent configurations result in movement trajectories through the interaction of emergent muscle forces and joint torques interacting with mechanical loads. The utility of the model-free control approach was demonstrated by Buchli et al. (2011), who used a robotic system in which: (1) a model-free reward function implemented trial-and-error learning and (2) a variable impedance controller allowed adaptability to different task and environmental properties. This type of control learns both reference trajectory and feedback gain schedules simultaneously, purely through experience and without the need for an *a priori* model of body and/or environmental dynamics. Model-free, trial-and-error learning may indeed be a sufficient mechanism for controlling both movement and posture, but in itself, it fails to account for evidence of internal representations of mechanical conditions that appear to allow both adaptation in—and generalization to—novel dynamic environments, such as applied force or inertial fields (Sainburg, 2015). In addition, a large amount of evidence indicates that the CNS takes inertial dynamics of body segments into account when making point-to-point reaching movements (Cooke and Virji-Babul, 1995; Sainburg et al., 1995; Ketcham et al., 2004), that sensory feedback is used to control evolving movement (Flanders et al., 1986; Cordo, 1990), and that human-object interactions are planned based on information about the physical properties and mechanics of the object (Dingwell et al., 2002; Cothros et al., 2006).

Motor selection and motor planning mechanisms involve optimizing costs, such as smoothness, accuracy, mechanical energy, etc., to produce energetically efficient trajectories (Flash and Hogan, 1985; Alexander, 1997; Todorov, 2004; Nishii and Taniai, 2009; Huang et al., 2012). This type of control requires a model of limb dynamics and a cost function in order to be able to find optimal control trajectories and feedback gain schedules. Optimal feedback control assumes that the CNS is able to find the optimal solution for any given task by allowing variability in task-irrelevant dimensions while constricting variability that affects task goals (Todorov and Jordan, 2002). While this type of control may provide the ideal solution to an engineered system, this is not necessarily how the human CNS behaves, probably due to constraints imposed by evolution. Therefore, it is likely that we define a range of task-specific costs with different gains dependent on task conditions, and which allows us to rely on a local minimum that is “good enough,” i.e., satisficing rather than looking for the “optimal” solution (Rosenbaum et al., 2001; De Rugy et al., 2012; Loeb, 2021). It is important to note here that neural control of movement is not “ideal” to begin with (i.e., our movements are not always the most energetically efficient or least erroneous choice), and any assumption of its ideal nature forms an incorrect basis for models of human movement. For example, (Gribble et al., 2003) showed that co-contraction of shoulder, elbow and biarticular muscles increased with reduced target size in order to improve movement accuracy, even though this was an energetically inefficient solution. The magnitude of co-contraction reduced over the course of learning, suggesting that internal models are formed by the CNS to regulate viscoelasticity of the musculoskeletal system by producing the necessary feedforward commands (Thoroughman and Shadmehr, 1999; Osu et al., 2002). Thus, we essentially balance a tradeoff between movement accuracy and efficiency.

We propose a hybrid model of motor control in which efficient movement is specified through the combination of control mechanisms that account for internal and environmental mechanics, are mediated by feedforward and feedback control circuits, and which provide for the ability to effectively achieve a stable posture at the end of movement (Sainburg, 2014). The hybridization of predictive and impedance control mechanisms has been shown to produce smooth movements that can quickly adapt to unexpected perturbations (Takahashi et al., 2001; Scheidt and Ghez, 2007; Yadav and Sainburg, 2014). The authors have separately modeled reaching using a serial hybrid model with a forward dynamic controller for specifying an initial trajectory based on environmental and task conditions, and a postural impedance controller for specifying a final equilibrium position (Scheidt and Ghez, 2007; Yadav and Sainburg, 2011). The serial hybrid model has been used to explain interlimb differences in reaching behavior related to the time of switch from trajectory control to impedance control, whereby the left hand switches from trajectory to impedance control early in the movement (Duff and Sainburg, 2007; Schabowsky et al., 2007). In contrast, the right hand’s advantage in controlling intersegmental dynamics arises from a later shift to impedance control, which allows time for sensory feedback to be integrated into online trajectory control. These two control schemes differ in terms of computational and metabolic costs, which suggests that both schemes must work together. We speculate that the two control schemes arose due to an evolutionary advantage for a division of labor between cerebral hemispheres.

## Lessons From Human Psychophysics

Even the most simple of actions, such as goal-directed reaching, appear to be implemented as a sequence of distinct control actions that specify movement trajectories and stabilized limb postures. Scheidt and Ghez (2007) designed two tasks that each emphasized one aspect of movement—control of ongoing trajectory (a slicing task) or final position (a point-to-point reaching task)—by providing knowledge of performance in the form of cursor feedback at the start or at the end of the movement. When participants adapted to a visuomotor rotation while completing either the reaching or slicing task, they only adapted to the specific feature of their performance that coincided with the provided feedback. Hence, participants only adapted the initial direction of their movements when provided (rotated) cursor feedback during movement but did not substantially adapt the final stabilized positions of those same movements. Similarly, they only adapted the location of their final stabilized hand position when provided (rotated) cursor feedback at the end of movement but did not substantially adapt the initial trajectory direction of those same movements. That is, adaptation of the spatial goal for movement did not transfer to the spatial goal for stabilizing the hand at the end of the same movement and *vice versa*. A second set of observations in these studies support the independence of control actions specifying the movement’s initial trajectory and final position. After practicing accurate point-to-point reaches from a start position to a target, participants overshoot the target dramatically when asked to make an out-and-back slicing movement that was to reverse direction in that same spatial target (Scheidt et al., 2011). They did so because the initial plan for movement in the slicing task, which was transferred from the reaching task, failed to account for the *absence* of increased joint impedance caused by increased joint antagonist coactivations in the neighborhood of the spatial goal during reaching but not slicing. Learning to terminate a reaching movement accurately should have allowed individuals to perform the slicing task accurately if they were guided by a common control mechanism driven by a single spatial goal. However, the experimental results indicated that different neural representations of the target position are formed to specify an initial trajectory and a final posture (i.e., that the control of trajectory and final posture are in fact distinct), that these control actions are typically performed sequentially during point-to-point reaching, and that transfer of a movement trajectory plan from one task to another related task (i.e., from reaching to slicing) does not automatically account for differences in joint viscoelasticity anticipated in subsequent phases of the action sequence.

Interlimb differences in task performance arise from hemispheric specialization for control actions regulating limb movement trajectory and final posture. Although a prominent view of handedness is that the dominant hand-hemisphere system is better at movement coordination and execution than the non-dominant side, the non-dominant hand has been shown to be superior at specific aspects of performance, such as stopping at a fixed position. In an experiment that required participants to reach from a fixed start position to multiple targets vs. from multiple start positions to a fixed target, it was found that the dominant hand’s performance was better for the former than the latter task, while the non-dominant hand’s performance was better on the latter task (Wang and Sainburg, 2007). The dominant system aims to minimize errors associated with intersegmental coordination and is advantageous in adapting to novel dynamic conditions (Sainburg, 2002). The non-dominant system is specialized for responding to unexpected perturbations and reducing deviations from achieving steady state postures (Mutha et al., 2012). Further evidence of hemispheric specialization for these distinct control processes arises from studies conducted on individuals with unilateral brain damage due to stroke. During a reaching task, right hemisphere damaged individuals were able to make fairly linear reaching movements toward a spatial target, but produced large errors in final position accuracy compared to neurologically intact controls and left hemisphere damaged individuals (Schaefer et al., 2009). In contrast, left hemisphere damaged stroke survivors produced significantly more curved movements, but were more accurate at the end position compared to right hemisphere damaged individuals. These results have been replicated in the contralesional arm of hemiparetic chronic stroke survivors as well (Mani et al., 2013).

Based on psychophysics, the evidence suggests that human motor control *satisfices* not *optimizes*. Our CNS is an evolved system that does not necessarily conform to an optimal engineered system. Evolution has played a crucial role in the selection of structure and function of the human nervous system, and this may help delineate between what is ideal and what is practical. Although our control system may not yield the most elegant solution from an engineering perspective, it has ensured survival as a species; hence, when studying neural control of movement, we must allow for the possibility that sensorimotor responses to changing and often unpredictable environmental conditions may not be optimal, in the engineering sense.

## Why Does Any of This Matter?

Hemispheric specialization has allowed for effective and efficient bimanual control (e.g., holding a slice of bread with one hand while spreading butter on it with the other). An understanding of hemispheric control mechanisms can allow the design of more personalized treatment strategies for individuals with brain deficits. For example, training the ipsilesional arm of left hemisphere damaged stroke survivors on tasks that promote movement coordination while training right hemisphere damaged individuals on tasks that require online corrections, stabilization, and stopping can be beneficial for achieving functional independence (Maenza et al., 2021). In addition, lateralized motor control processes can affect the strategies employed for retraining the less-impaired arm (Sainburg and Duff, 2006) and impact functional outcomes differently in left and right hemisphere damaged stroke survivors (Jayasinghe et al., 2020). Such insights promise benefits of personalized therapeutic and compensatory interventions for chronic stroke survivors.

Stopping, in particular, is a fundamental aspect of movement that is complex and deserves more attention than it currently receives. Metabolic costs of stopping may be different from those of trajectory control, and future work may be able to address how this impacts movement strategies. A recent study from our lab showed that stabilizing behavior was similar between stroke survivors and neurologically intact adults during a mechanically coupled bimanual task (Jayasinghe et al., 2021) even though previous work using unilateral tasks have shown performance deficits in stroke survivors. There is clearly more to the story of stabilization than meets the eye, and future work can focus on different types of stabilization tasks—online correction, staying in a fixed position during perturbation to the same hand, bimanual stabilization, etc., to form a deeper understanding of this complex phenomenon and its specialization within the brain.

## Author Contributions

SALJ, RAS, and RLS interpreted findings and wrote the original draft of the manuscript. All authors contributed to the article, reviewed and approved the submitted version.

## Conflict of Interest

The authors declare that the research was conducted in the absence of any commercial or financial relationships that could be construed as a potential conflict of interest.

## Publisher’s Note

All claims expressed in this article are solely those of the authors and do not necessarily represent those of their affiliated organizations, or those of the publisher, the editors and the reviewers. Any product that may be evaluated in this article, or claim that may be made by its manufacturer, is not guaranteed or endorsed by the publisher.
